# Splitting chemical structure data sets for federated privacy-preserving machine learning

**DOI:** 10.1186/s13321-021-00576-2

**Published:** 2021-12-07

**Authors:** Jaak Simm, Lina Humbeck, Adam Zalewski, Noe Sturm, Wouter Heyndrickx, Yves Moreau, Bernd Beck, Ansgar Schuffenhauer

**Affiliations:** 1 KU Leuven, ESAT-STADIUS, Kasteelpark Arenberg 10, 3001 Heverlee, Belgium; 2 Medicinal Chemistry Department, Boehringer Ingelheim Pharma GmbH & Co. KG, Birkendorfer Str. 65, 88397 Biberach an der Riss, Germany; 3 Amgen Research (Munich) GmbH, Staffelseestraße 2, 81477 Munich, Germany; 4 Novartis Institutes for BioMedical Research, Novartis Campus, CH-4002 Basel, Switzerland; 5 Janssen Pharmaceutica N.V., Janssen Pharmaceutica, Turnhoutseweg 30, 2340 Beerse, Belgium

**Keywords:** Cross-validation, Train-test-split, Federated machine learning, Leader follower clustering, Sphere exclusion clustering, Locality-sensitive hashing, Scaffold tree, Scaffold network, ChemFold

## Abstract

With the increase in applications of machine learning methods in drug design and related fields, the challenge of designing sound test sets becomes more and more prominent. The goal of this challenge is to have a realistic split of chemical structures (compounds) between training, validation and test set such that the performance on the test set is meaningful to infer the performance in a prospective application. This challenge is by its own very interesting and relevant, but is even more complex in a federated machine learning approach where multiple partners jointly train a model under privacy-preserving conditions where chemical structures must not be shared between the different participating parties. In this work we discuss three methods which provide a splitting of a data set and are applicable in a federated privacy-preserving setting, namely: a. locality-sensitive hashing (LSH), b. sphere exclusion clustering, c. scaffold-based binning (scaffold network). For evaluation of these splitting methods we consider the following quality criteria (compared to random splitting): bias in prediction performance, classification label and data imbalance, similarity distance between the test and training set compounds. The main findings of the paper are a. both sphere exclusion clustering and scaffold-based binning result in high quality splitting of the data sets, b. in terms of compute costs sphere exclusion clustering is very expensive in the case of federated privacy-preserving setting.

**Supplementary Information:**

The online version contains supplementary material available at 10.1186/s13321-021-00576-2.

## Introduction

In machine learning it is good practice to split the data set in multiple folds to be used exclusively for training, hyperparameter optimization and final performance evaluation, often in combination with cross-validation [1]. The most straightforward approach for fold splitting is a random split, however this is not ideal for structure-activity models. An early observation with quantitative structure-activity relationship (QSAR) models is the so-called Kubinyi paradox: models with the best cross-validated performance metrics were the ones worst performing in a prospective setting [2, 3]. The reason for this is that many QSAR data sets contain only a limited number of chemical series, however in the prospective use case the model should be applicable also to other structures not belonging to this chemical series. Lombardo et al. [4] replaced the leave-one-out cross-validation by a leave-class-out cross-validation protocol, where entire structure classes were left out. This avoids the leakage of training data into the test set by having close structural analogues of the training structures in the test set (series effect). In large-scale machine learning endeavors human class labeling as done by Lombardo et al. is not practical, and typically less folds are used than there are structural classes in the data set. Clustering can in this situation replace human classification, and whole clusters are assigned to one fold [5] to ensure that the test fold is structurally reasonably dissimilar to the training and validation fold. Another approach to asses model performance realistically is the time-split cross validation, where data is split according to the time-stamp it was generated [6]. Like Martin et al. Sheridan stated that random splits of data sets for machine learning lead to overoptimistic performance assessments. Also other domains of machine learning have recognized the problem arising from assessing model performance based on random splits [7].

### Fold splitting in sparse multi-task settings

**Fig. 1:**
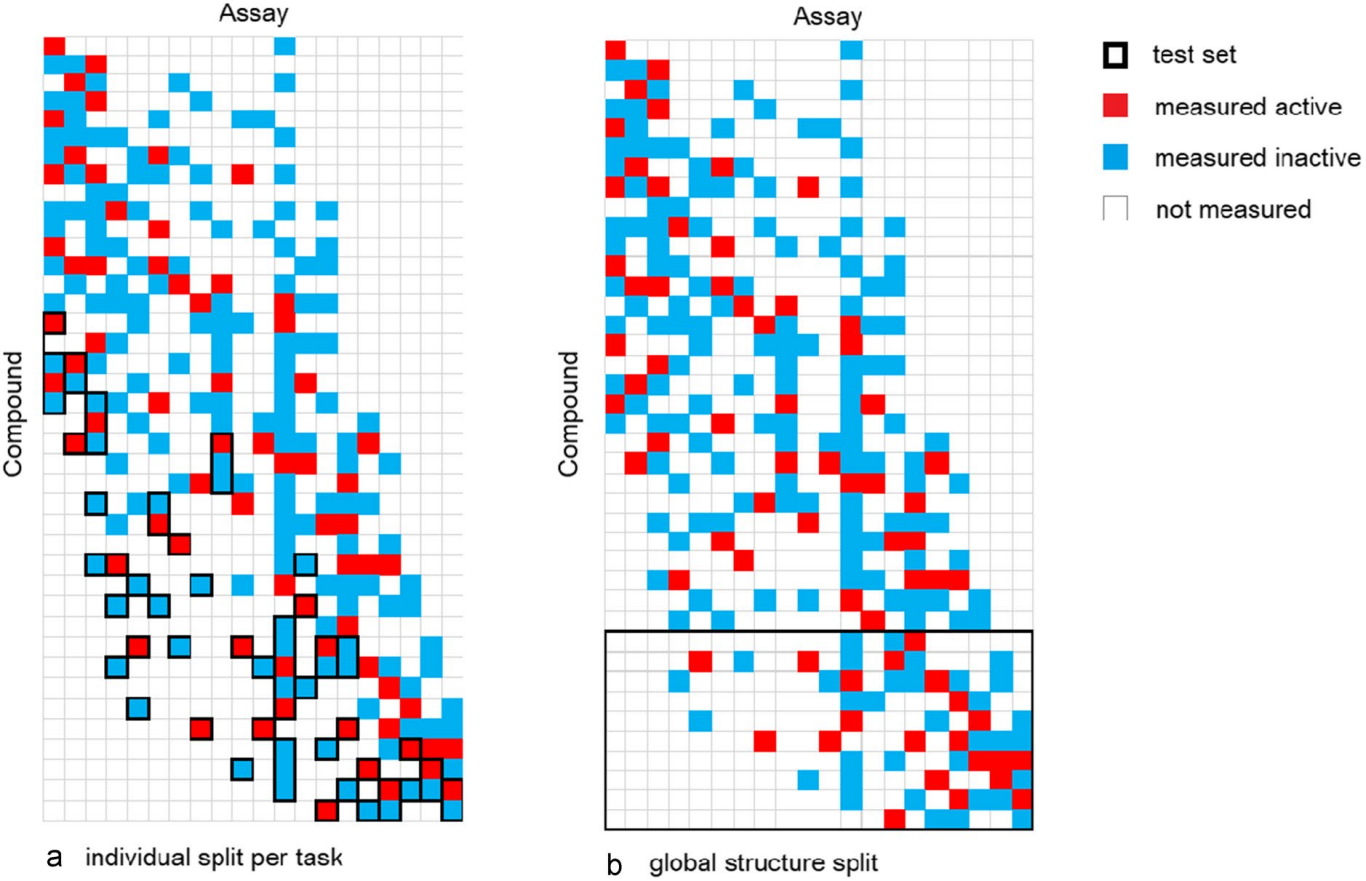
Fold splitting in sparse multitask setting. For fold splitting in sparse multitask setting there are two approaches **a** Fold splitting is done on a per task level. For each task the observations are assigned to the test fold independently. In this setup, there are compounds which are for some assays in the test fold and for others in the training set. **b** Fold splitting on the whole compound space, where a compound with all its measurements is assigned to one fold

While the fold splitting in a single task setting with the methods mentioned above is straightforward, sparse multi-task settings, as encountered when modelling the structure activity matrix of a larger pharmaceutical company, pose an additional challenge. There are many diverse assays, but each of the assays has measurements only for a small fraction of compounds that are included in total, in other words the assay-compound activity matrix is sparse. The situation is illustrated in Fig. 1. In such a situation fold-splitting can be done for each task (assay) independently (Fig. 1a). This allows full control over the fraction of compounds assigned to each fold, and thus ensures that a fixed fraction of compounds gets assigned to the test set for each assay. The downside of this approach is, that a compound may for some tasks end up in the training set and for others in the test set. Thus, compound structure information is leaked from the training into the test set, which is then not anymore fully independent. Therefore machine learning performance metrics obtained from this type of fold splitting are more indicative of the expected performance for filling in the sparse compound activity matrix than for predicting assay outcomes in structurally distinct and novel chemical structure spaces. The alternative is fold splitting in the whole compound space. Here, a compound is assigned with all its measurements to one fold (Fig. 1b). If the fold splitting in compound space is executed through clustering, then this can ensure that the test fold is clearly independent and structurally distinct from the training set. Therefore it can be expected that performance figures generated under this fold splitting regime are indicative for the prediction performance on novel structures. The downside of this approach is that it cannot be guaranteed anymore that a fixed split ratio between training and test folds is maintained. As a consequence, the resulting split-ratios in this approach need to be carefully monitored. An optimal train test fold split in the compound domain needs to reconcile the two competing objectives of separating the train and test folds in the chemistry space on one hand while maintaining a reasonable split of each of the tasks data between train and test set on the other hand. A time-gated split [6] under this paradigm would have to rely on the compound registration date as a time stamp independent of the assays. Given that many assays are run only for a limited period in time, it is impossible to find a cutoff-date which is suitable for all assays at the same time. For many assays, a time-gated split on the whole compound domain will lead either to an empty training or an empty test set. In this article we will focus on whole compound domain splits that will allow to assess the prediction performance on novel compounds, even if this makes using time-gated splits practically impossible.

### Federated machine learning

In federated machine learning, the data is not centralized at one server but stored locally by the individual parties owning it. This enables the preservation of privacy, ensuring that no other party than the owner can access the data. The communication between partner servers is limited to secure updates of model parameters [8]. This technology allows training machine learning models on data from multiple owners, that cannot be co-located centrally for privacy or intellectual property reasons. In this way the data owners benefit from a larger training set and can expect a higher predictive performance of their models without putting their data at risk. In the area of drug discovery, the chemical structure data and the corresponding activity labels are sensitive information with respect to intellectual property and must kept under the control of the owning company at all times. Likewise similarity information, namely which structures of one partner are structurally similar to those of other partners is sensitive, and therefore computation of complete, cross-partner similarity matrices (which is the basis for many clustering approaches) is not possible.

**Fig. 2:**
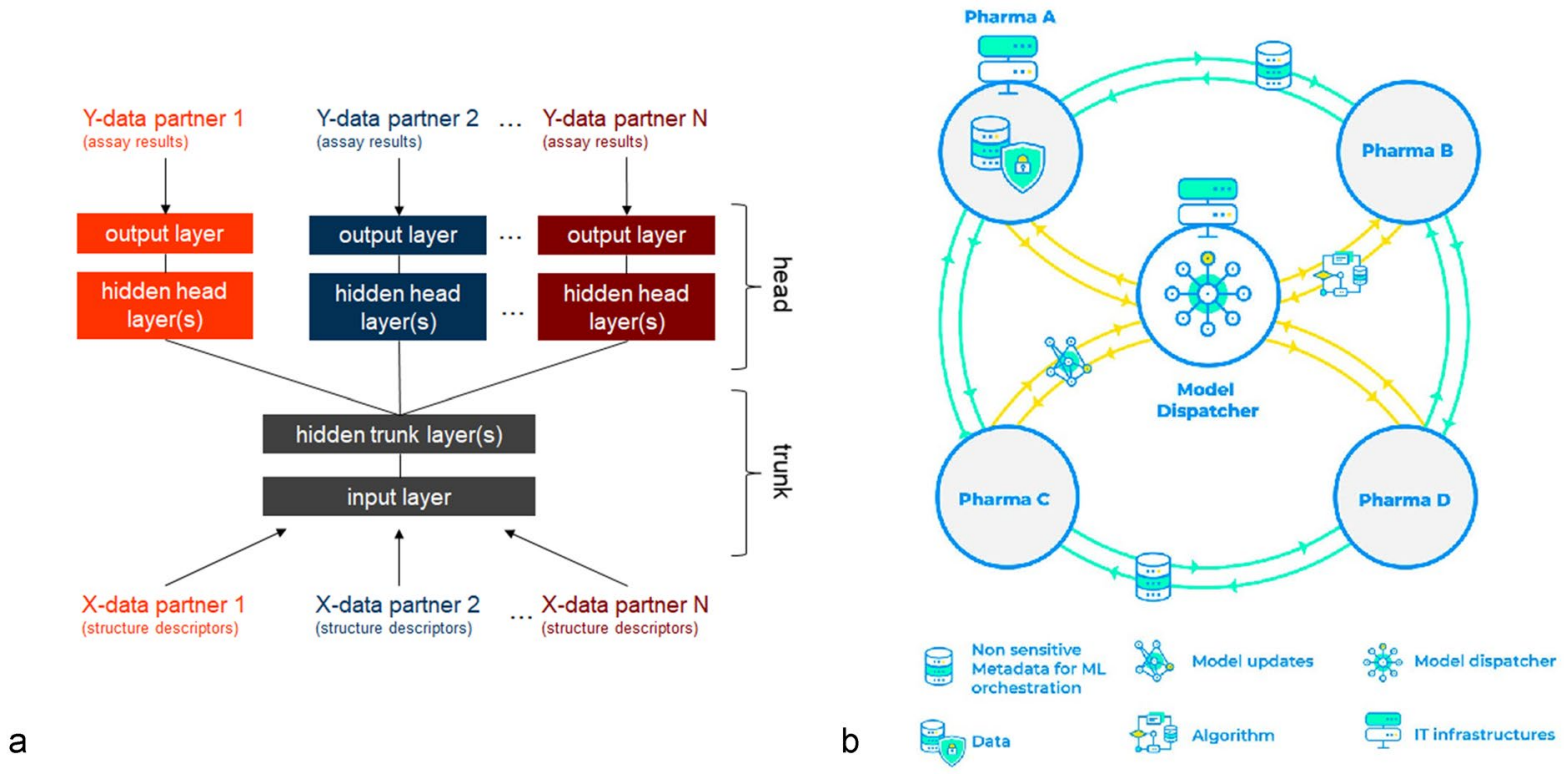
Federated Machine learning scheme in MELLODDY. **a** MELLODDY uses a feed-forward neural network, which is divided in a trunk and head part. The trunk is jointly trained by all partners, using secure aggregation of parameter updates at each iteration. The head part is individual to each pharma partner, an thus allows each partner modelling their own assays without mapping to the other partner’s assays. The common trunk enables transfer learning between the tasks of different partners. **b** The technical architecture of MELLODDY. Each partner maintains a node on a cloud platform. An additional model dispatcher node aggregates the trunk updates, but is prevented by the secure aggregation protocol from accessing these updates itself

In MELLODDY (**M**achin**E** **L**earning **L**edger **O**rchestration for **D**rug **D**iscover**Y**, [9]) a multi-task structure-(bio)activity and property model is trained by using the data from 10 pharmaceutical companies. The pharma partners convert their structures into standardized fingerprint representation using the shared MELLODDY TUNER^1^ protocol, and then add their assays as prediction tasks for a joint feed-forward neural network. This network is split in a trunk part, which is jointly trained by all partners, and a head part where each company trains it own head. (see Fig. 2a). In contrast to a typical federated learning project, where all partners train a model on a shared set of tasks, here the pharma partners train their individual assay tasks, using a hidden representation jointly trained by all partners. The trunk of the model computes the common hidden representations from all input structure descriptors, whereas each head computes the prediction outputs for only the assays owned be a given partner. The reason for choosing this approach is as follows: different assays on the same target in general do not yield quantitatively identical results, unless also their experimental setups are closely similar. Such a close matching of experimental conditions is not only technically challenging, but also prohibitive from a privacy point of view, as pharma partners consider their target and assay portfolio as confidential information which cannot be shared. However, a benefit can be expected by several means, a better hidden representation based on a broader chemical and bioactivity space coverage, better performance for tasks through transfer learning between related tasks either within a company (multi-task benefit) or throughout different companies (federated benefit) and an extended applicability of tasks via an enrichment of the bioactivity matrix based on transfer learning between related tasks leading to more robust models with greater prospective applicability. MELLODDY will perform machine learning on an unprecedented amount of training data and, even in absence of mapping of assays onto common tasks, synergies in the joint latent representation resulting from an partial overlap of the partners chemical structure space [10, 11] and target space are expected to lead to a prediction performance gain.

Technically MELLODDY relies on the Substra framework [12], a federated machine learning platform (see Fig. 2b). In order to train the shared network trunk, each partner hosts a copy of this part of the model. After each partner has updated his trunk by training on a mini-batch of own data, the trunk weight updates are aggregated through a secure protocol similar to [13] making it impossible to back-track contributions to individual partners. Then, each partner continues training the model with the next mini-batch of its training data followed by another aggregation step. This is continued until the training of the model is completed.

While privacy-preserving, federated machine learning offers the opportunity to improve model performance, it imposes constraints on the protocols that can be used for fold splitting. As the data cannot be aggregated in one place, only two types of fold splitting protocols are feasible: (1) protocols that calculate fold assignment at each partner site independently from other partners, and (2) clustering protocols that can be run in a federated setup and do not require co-location of the data. One possibility for (1) is to execute the fold splitting on a per task basis, as done in [5]. Here we are aiming for a fold split in the entire structure domain across all partners, where a chemical structure with all its activity data is allocated to one fold, in order to obtain a realistic assessment of the ability of a model to predict activity information for novel compounds without any experimental results. Consequently, all protocols have to fulfill the minimum requirement that identical structures are allocated to the same fold consistently across all partners, should a compound exist in the data set of more than one partner. In this article, several fold splitting methods compatible with the constraints of privacy preservation are described, and their impact on machine learning performance is compared. As a baseline random splits were considered, despite the fact that these are not trivial to execute in a federated setting, under the constraint of consistent mapping of identical structures.

Three fold splitting methods applicable both in traditional and federated privacy-preserving settings are investigated: a. locality-sensitive hashing (LSH), b. sphere exclusion clustering, c. scaffold-based binning (scaffold network) and compared to a random fold splitting. Besides random splitting, two of the methods described in this article - locality-sensitive hashing and scaffold-based binning - can be executed at each partner autonomously during data preprocessing and do not require any federated computation. The third method sphere exclusion clustering is a clustering protocol, that requires federated execution, but is compatible with the privacy preservation constraints. All fold splitting methods were executed on 5 large-scale structure-activity data sets: a data set each from four pharmaceutical companies participating in MELLODDY (Amgen, Boehringer Ingelheim, Janssen, and Novartis) covering at least 50% of the structure-activity data warehouse of the company, and in addition a curated data set derived from ChEMBL [14]. Time-Split cross-validation [6] is not considered for the reason discussed above.

## Materials and methods

In this section we, firstly, introduce the data sets we will employ for empirical comparisons of different splitting methods. Secondly, we give a detailed overview of these methods. Additionally, the implementation of each method in a federated privacy-preserving setting is covered.

In what follows we assume that all the parties share a common pipeline to standardize molecules, including handling of, e.g., ions and tautomerization. The pipeline we will use in this work is described in the next section.

### Data sets

A public data set was gathered from ChEMBL version 25 [14] and preprocessed using code provided by Iktos, a MELLODDY participant developing user-friendly artificial intelligence algorithms for drug design.^2^ Afterwards, the public data were prepared by MELLODDY TUNER.^3^ MELLODDY TUNER is a RDKit-based pipeline to standardize and prepare structure-activity data for machine learning and covers the privacy-preserving federated learning application. The standardization protocol contains handling of charges, stereochemisry, tautomerism, isotopes and sanity checks, e.g., number of heavy atoms, proper SMILES. Subsequent to standardization a canonical SMILES as well as an extended connectivity fingerprint (ECFP) [15], i.e., Morgan Fingerprint as implemented in RDKit, are calculated and used as descriptors in model training. In this work a 32k folded ECFP6 was utilized. MELLODDY TUNER also covers formatting activity data, e.g. handling of duplicate entries. For descriptor-based fold splitting methods, e.g., LSH and sphere exclusion clustering, it is crucial to ensure a canonical descriptor, i.e., identical compounds lead to an identical fingerprint. To this end, identity has to be defined, e.g., in this work different salt forms of the same parent compound are considered and treated as identical.

The public data set was derived from more than 508,000 chemical structures and the final set (hereafter referred to as ChEMBL set) consists of 2978 assays and 4083 binary classification tasks. This processed public data set [16] is available online.^4^

The private data sets from four MELLODDY partners (Amgen, Boehringer Ingelheim, Janssen and Novartis) participating in this study consist of 2000-12000 assays, primarily bio-activity assays measured in concentration response mode along with ADME/physchem-related assays. Each assay was transformed into 1-5 classification learning tasks by applying different cutoffs on the assay readout. For example an assay yielding $IC_{50}$ values, could be once cut at a threshold of 1 M and once at 10 M. In this case in the first of the two tasks all samples with an $IC_{50}$ below 1 M would be considered as active, whereas in the second task all samples with an $IC_{50}$ below 10 M would be considered as active. The choice of the activity thresholds was left to the individual partners. Otherwise the private data sets were prepared in a consistent way throughout all four partners using MELLODDY TUNER, in analogy to the public set. It is noteworthy, that the tasks are not mapped between the partners and that each partner included at least 50% of their internal volume of concentration-response data.

### Random split

Random splitting is common practice in non-federated modelling, where one randomly assigns each compound, together with its measurements, to a fold. However, as compound data sets include series of compounds, random splitting will result in each series being present in **several folds**, and thus, also be present both in training and test set. Therefore, the final performance metrics computed on the test set are commonly expected to look unrealistically optimistic because in real-world applications researchers are interested in predicting compounds from series that were not seen during the training.

#### Federated implementation

To implement the federated random split under the constraint of consistent mapping of identical structures without online communication a hashing function can be used, for example SHA256 [17]. Hashing functions map their input consistently to an integer space (e.g. 256-bit) in a pseudo-random way. The output of this hashing function can then be used as a seed to a classic random number generator and generate a number from 1 to $N_{\mathrm {folds}}$ in order to obtain the fold assignment, where $N_{\mathrm {folds}}$ is the number of folds. If it is desirable that only the partners are able to compute the fold assignment, they can share a common secret, that gets appended to input of the hashing function.

If all partners agree on a common molecule standardization pipeline and use this to generate a unique representation for each structure (for example a canonical smiles) and then use this as input to the hashing procedure described above, the result is a pseudo-random but deterministic mapping of each individual chemical structure to a fold, which can be reproduced independently by the partners.

### Sphere exclusion clustering

A general idea for removing the overoptimistic bias of random splitting is to first assign compounds into series-like groups and then distribute each group randomly to one of the folds. While groups can be formed in various ways, a popular approach is to use sphere exclusion clustering (also known as Taylor-Butina or leader-follower clustering, short LFC) [18–20]. A target distance threshold $t_\mathrm {Tc}$ must be provided that defines the size of the clusters in terms of the Tanimoto distance of some fingerprint. In our study we used the ECFP [15] fingerprints generated by MELLODDY Tuner as described above with $t_\mathrm {Tc}=0.6$ as the Tanimoto distance cutoff for creating clusters.

Given the cutoff and fingerprints of the compounds the sphere exclusion clustering proceeds as follows:

1. Initialize an empty set of cluster centers.
2. Randomly pick a compound *c* that has not been yet processed.
3. Compute Tanimoto distances of the fingerprint of *c* to all of the existing cluster centers.
4. If the distance to the closest cluster center is less than or equal to $t_\mathrm {Tc}$ then assign *c* to that cluster. Otherwise create a new cluster with *c* as its center.
5. Repeat steps (2)-(4) as long as there are non-assigned compounds left.

After the initial assignment has been completed it is useful to re-assign all compounds to their closest clusters. This step corresponds to the re-assignment step in the *k*-means clustering. The computational complexity of the sphere exclusion algorithm is $O(N_\mathrm {clusters} N_\mathrm {compounds})$ and in non-federated settings can handle millions of compounds.

Finally, for each cluster we choose a random fold from 1 to $N_\mathrm {folds}$ to which we then assign all of the compounds in the cluster.

#### Federated implementation

The federated setting requires the partners to agree on a common molecular fingerprint generation protocol, which is typically the same common protocol used to generate the descriptors for the federated model training itself. In the federated setting one should take care that the cluster centers, required for distance calculations, have to be hidden from the parties. This means that straight-forward implementations are not possible and one should use cryptographically secure protocols, such as secure multi-party computation (SMPC) [21].

Using SMPC each partner can add its compound library to an encrypted space, where only cryptographically agreed computations are allowed to happen. The main idea in SMPC is to use secret sharing protocols, e.g., Shamir’s Secret Sharing, SPDZ [22] and SCALE-MAMBA. In these methods there is no third party who has access to the private data but instead each partner has a piece of the share of the data (i.e., compound descriptors) and the computation is done in encrypted form, such that only the final output is revealed to the parties. However, it should be noted that SMPC-based methods have very high computational and communication overhead, as they employ advanced cryptographic methods.

A general SMPC algorithm for a sphere exclusion clustering-like method works as follows:

1. Each party computes folded ECFP features for its compounds, e.g., 1024 bits.
2. Each party creates a secret sharing of the fingerprints adding them to the SMPC system.
3. The secretly shared fingerprints are pooled and randomly shuffled into a single list. The shuffling may only reveal each party’s compound locations to itself.
4. The main iteration loop of sphere exclusion clustering is executed:
  - The next compound is picked from the shuffled list and the distance of its fingerprint to all existing cluster centers is computed, the results are kept in secret sharing (*i.e.*, undisclosed).
  - Minimum distance $d_i$ is computed over the distances, also kept in secret sharing.
  - This distance $d_i$ is compared against $t_\mathrm {Tc}$ and the resulting bit $d_i > t_\mathrm {Tc}$ is revealed to all parties.
  - If the bit is 1 the secret shares of the *i*-th compound are appended to the cluster center list.
  - If there are still compounds left then continue from step 4a.
5. Finally, for each compound we compute the distances to all cluster centers, and find the center *j* with the smallest distance, in secret. Then the center value *j* is revealed to the party that owns the compound.

These steps outlined above are likely to be too slow for data sets with millions of compounds. To improve the speed it is possible to run in advance a SMPC version of k-means clustering with *k* equal to for example 100, which will create *k* clusters of compounds. Finally, the cluster assignments can be revealed and then for each cluster the sphere exclusion clustering can be executed in a computationally feasible manner.

As it can be seen above, this protocol is quite complex. Implementing it with the required level of security is a substantial effort. For large-scale modeling that uses millions of compounds and thousands of clusters, we expect the SMPC to be still quite heavy in computation. However, for smaller scale federated learning setups it might be a feasible and attractive option.

As in this paper we are focusing on large-scale federated learning settings and are interested to find fast and easy-to-use solutions, we will next introduce two approaches that are well suited to the federated setup. The hope is that we can achieve the same quality of split as sphere exclusion clustering but with much lower implementation and computation effort. Thus, before investing resources in a federated implementation of sphere exclusion clustering, a non-federated implementation was analyzed by four pharma companies independently and compared to easier to implement folding schemes.

### Locality-sensitive hashing

Similarly to the sphere exclusion clustering the locality-sensitive hashing (LSH) tries to put similar compounds into the same bin (binning), hence, reducing the series effect [23]. However, in contrast to the clustering approach, in LSH each compound and data set is processed independently, thus, making it well-suited for federated implementation.

The main idea of LSH is to bin compounds according to a subset of the descriptor, e.g., ECFP. Specifically, in our setting we consider picking *N* highest entropy bits (e.g., $N=16$) over the molecules of a data set. Note that having higher entropy, for a binary fingerprint, means to have frequency closer 0.5. Thus, we select the features whose frequency in the data set is the closest to 0.5. Each compound is then characterized by its *N* binary features and added to the given bin. In this study, where $N=16$ was chosen, there will be at most $2^{16}=65536$ bins, each bin containing compounds that have the same identical values for these 16 binary features. The property of such binning is that similar compounds have high probability to be assigned to the same bin. Each bin will then be randomly assigned to a fold,

Finally, compounds in each bin are all assigned to a same randomly generated fold (from 1 to $N_{\mathrm {folds}}$)

#### Federated implementation

In the federated setting, the LSH binning requires that all parties agree on a common subset of the fingerprint features. Therefore, we use a public data set, such as ChEMBL, to calculate the fingerprint feature frequencies and choose *N* features whose frequency is closest to 0.5. A large overlap is expected between the public *N* highest entropy bits and the private ones.

Each party subsequently proceeds to independently calculate the bins for all of its compounds using the selected features derived from the public data set. Finally, the hashing procedure described above for the federated random split is used to map each bin to a folds in a pseudo-random fashion, using an integer representation of the bin as input to the hashing function. The difference between LSH and the random split is, that in case of LSH a group of several to some degree similar compounds shares the same bin, and is thus assigned to the same fold, whereas in the random split each compound produces its unique input for the hashing procedure.

### Scaffold-based binning

Scaffolds are a chemically intuitive way of grouping chemical structures. The first approach to automatically determine scaffolds consisted of pruning all acyclic sidechains from the core scaffold (“Murcko”-scaffold) [24]. A fold split according to these scaffolds has been described by [25] where it has been demonstrated that this type of split leads to much less optimistic model performance assessments than a random split. For practical purposes this scaffold definition is often too granular, as, for example, the addition of a simple phenyl group to an existing scaffold creates a new scaffold. This can be remedied by pruning this initial “Murcko”-scaffold further by iterative removal of single rings, thereby removing with preference more peripheral, chemically less characteristic rings [26]. This turns the flat scaffold classification in a hierarchical classification tree. The tree levels that correspond to scaffolds with fewer rings are more generic. This approach can be used for structure-activity relationship investigations [27] and has been further generalized by the scaffold network [28], where in contrast to the scaffold tree multiple decomposition alternatives are considered, meaning that at each hierarchy level a structure can be associated to more than one scaffold. The scaffold network algorithm was implemented in RDKit version 2020.03 [29]. In contrast to the original scaffold network implementation, the RDKit version in its default configuration does not dissect fused, bridged or spiro ring systems, with the effect that non-intuitive dissections like, for example, the dissection of a steroid ring system are prevented.

**Fig. 3:**
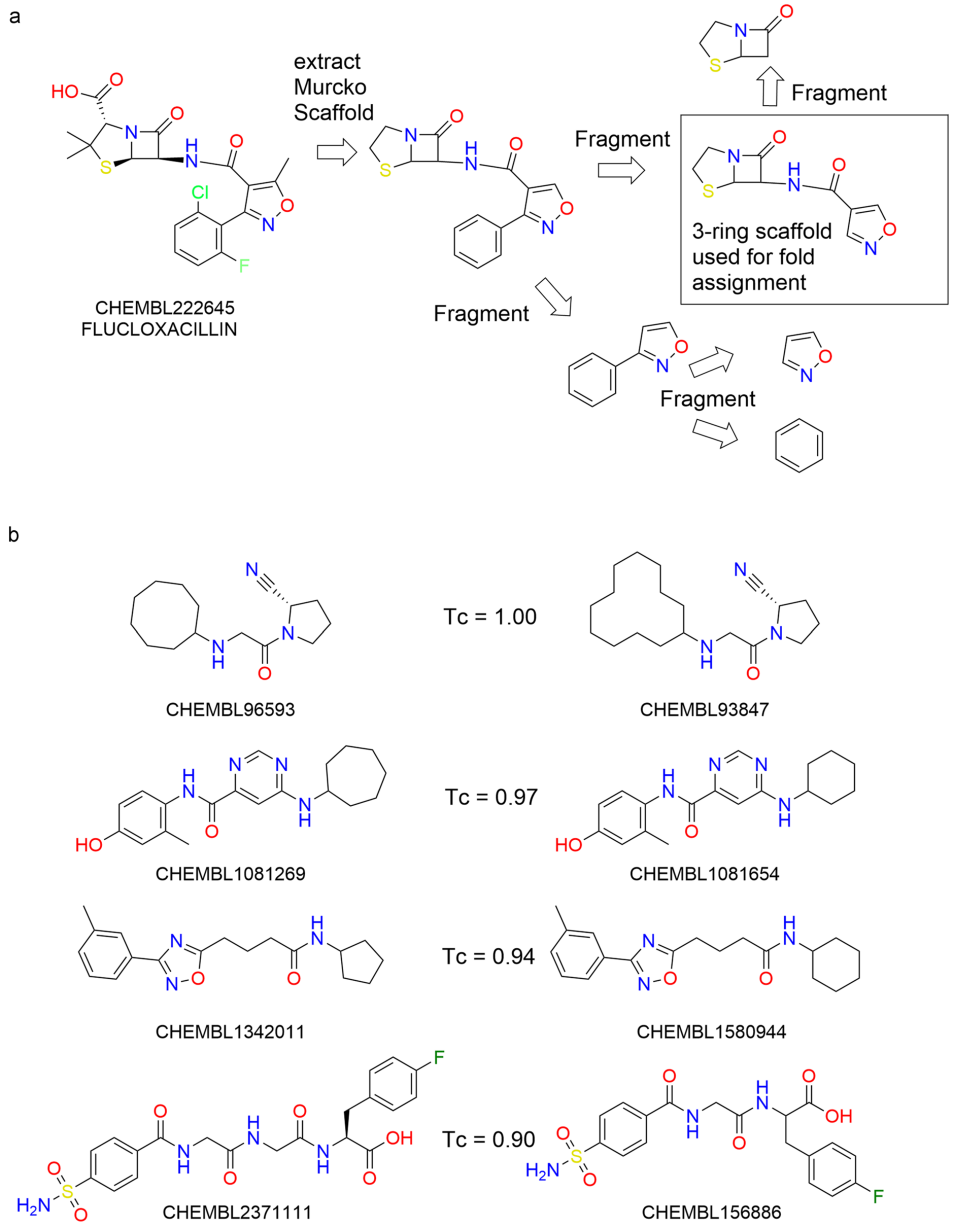
Scaffold based fold splits **a** The scaffold extraction procedure is illustrated on the example of flucloxacillin. **b** Examples of highly similar compounds from ChEMBL by means of Tanimoto similarity (Tc) of a fingerprint (ECFP6 32k folded) that are assigned to different scaffolds

For this study the RDKit implementation was used due to the wide distribution and usage of this toolkit. The RDKit implementation has features for both retaining of attachment point information as well as generating abstract scaffolds with generic atoms and bond types. Both of these features were deactivated for this study. In addition, the multi-class classification approach of scaffold network is not suitable for fold splitting, where each compound must be assigned to one class only. Thus it was necessary to post-process the output. For practical purposes in medicinal chemistry, scaffolds with three rings often provide a useful level of granularity [30]. Therefore, from the scaffolds generated by the RDKit scaffold network implementation all scaffold with three rings were selected. In case no such scaffold exists, the scaffolds with the number of rings closest to 3 were selected. If more than one 3-ring scaffold exists, the original scaffold tree rules from [26] were applied for further prioritization in order to select a single scaffold. An example is shown in Fig. 3a.

It should be kept in mind that in contrast to LSH and sphere exclusion clustering this scaffold-based approach is a heuristic one which is independent from an underlying fingerprint. There can be in principle two structures which have highly similar or even identical fingerprints, but different scaffolds, especially through ring-size or linker length extension. Example compound pairs from ChEMBL which end up in different folds, despite high Tanimoto similarity (Tc) in the ECFP6 32k folded fingerprint, are shown in Fig. 3b.

#### Federated implementation

To implement the federated scaffold-based binning, the scaffold obtained in the described way is translated into a SMILES string which is then, in analogy to LSH, submitted to the hashing procedure described above in order to obtain a consistent, pseudo-random fold assignment for each scaffold. As all acyclic structure have an empty scaffold smiles, this leads to the effect that all acyclic molecules are hashed to the same fold. Like LSH, scaffold-based fold assignment is fully deterministic and can be run locally at each pharma company.

### Evaluation criteria

The fold splitting methods were evaluated according to three criteria (1) distance of the test compounds to the training set, (2) label and data imbalance, (3) bias in prediction performance. The first criterion describes how well the training and the test set are separated in the fingerprint descriptor space. Pairs of closely analogous compounds should be contained in one fold to the largest possible extent and not be split across different folds. This is a more loose criterion than the typical evaluation criterion for clustering, where an overall high intra-cluster similarity is aimed at. To asses this, pairs of molecules were sampled from the whole data set and grouped into 10 equidistant bins based on the Tanimoto similarity of their fingerprints: 0.0-0.1, 0.1-0.2 ... 0.8-0.9, 0.9-1.0. For each of the similarity bins, the fraction of pairs where both compounds originate from the same fold was computed. From a purely random fold split one would expect a fraction of $1/N_\mathrm {folds}$ pairs being allocated to the same fold irrespective the similarity bin. In this study 5 folds have been generated, thus the baseline probability of a pair being allocated to the same fold is 0.2. For our splitting methods we expect that the fraction of intra-fold pairs increases with increasing similarity, and should approximate 1.0 for similarity values close to identity.

While concentrating pairs of close analogues in one fold, distribution of overall observations across the folds for each task should be as homogeneous as possible. Likewise the fraction of positively labeled observations for each task across the folds should be homogeneous. This is a difficult to achieve objective, as the y-matrix of observations in multi-task QSAR models is typically very sparse, and for many tasks only few structural classes of compounds have been measured in the underlying assay. Even if multiple series have been tested in an assay, it may still be the case that the positively labeled “active” compounds result only from a small number of chemical series. In this case methods aiming to assign complete chemical series to one fold may assign the majority of actives to one or two folds. As a consequence, the fraction of actives per fold will differ between the folds. This can be quantified by calculating the standard deviation of the fraction of actives across the folds for each task. In such a situation either the model performance may suffer, if the active sample are predominantly in the test set, or the accuracy with which the performance can be quantified suffers from a too small number of active compounds in the test set. In a single task setting, it is possible to mitigate these effects by assigning the individual clusters to each fold in such a way that the balance of labels is maintained. As discussed in the introduction, this is not possible in a sparse multi-task setting, without introducing information leakage from the training to the test set. In the compound domain splits described here no mitigation for the data and label imbalance between folds was applied.

Finally the most relevant objective is the impact of the fold splitting on the machine learning. For this purpose the performances of a machine learning model trained using different fold splitting methods are compared. It is expected that more stringent fold splitting methods with a better separation between test and training data will achieve a lower performance. This lower performance will however reflect more closely the performance that can be expected under realistic real-world medicinal chemistry applications, compared to the **overoptimistic** performance readouts from random fold splits.

Unless stated otherwise, aggregated results over individual pharma partners and all tasks are reported.

### Training process

First the data was clustered according to the respective method and then split into training, validation and test folds, 60-20-20 ratio, i.e, nested cross-validation. For each method the validation set was used to find the hyperparameters that maximized its performance on mean AUPR across the tasks. SparseChem^5^ a Python package for machine learning models for physicochemical applications was used to generate single partner feed-forward neural networks predicting multiple binary classification tasks.

For the hyperparameter optimization we scan the parameters given in Table 1.

**Table 1:** Hyperparameter grid used in the optimization

Hyperparameter	Set of values
Hidden sizes	[1200], [1600], [2000], [3000], [1600, 1600]
Dropout	0.4, 0.5, 0.6, 0.7
Weight decay	1E−5, 1E−6

The number of training epochs was fixed to 25. The learning rate was 0.001 and it was reduced at epoch 20 by 0.3 times.

## Results and discussion

### Similarity of test and training set

**Fig. 4:**
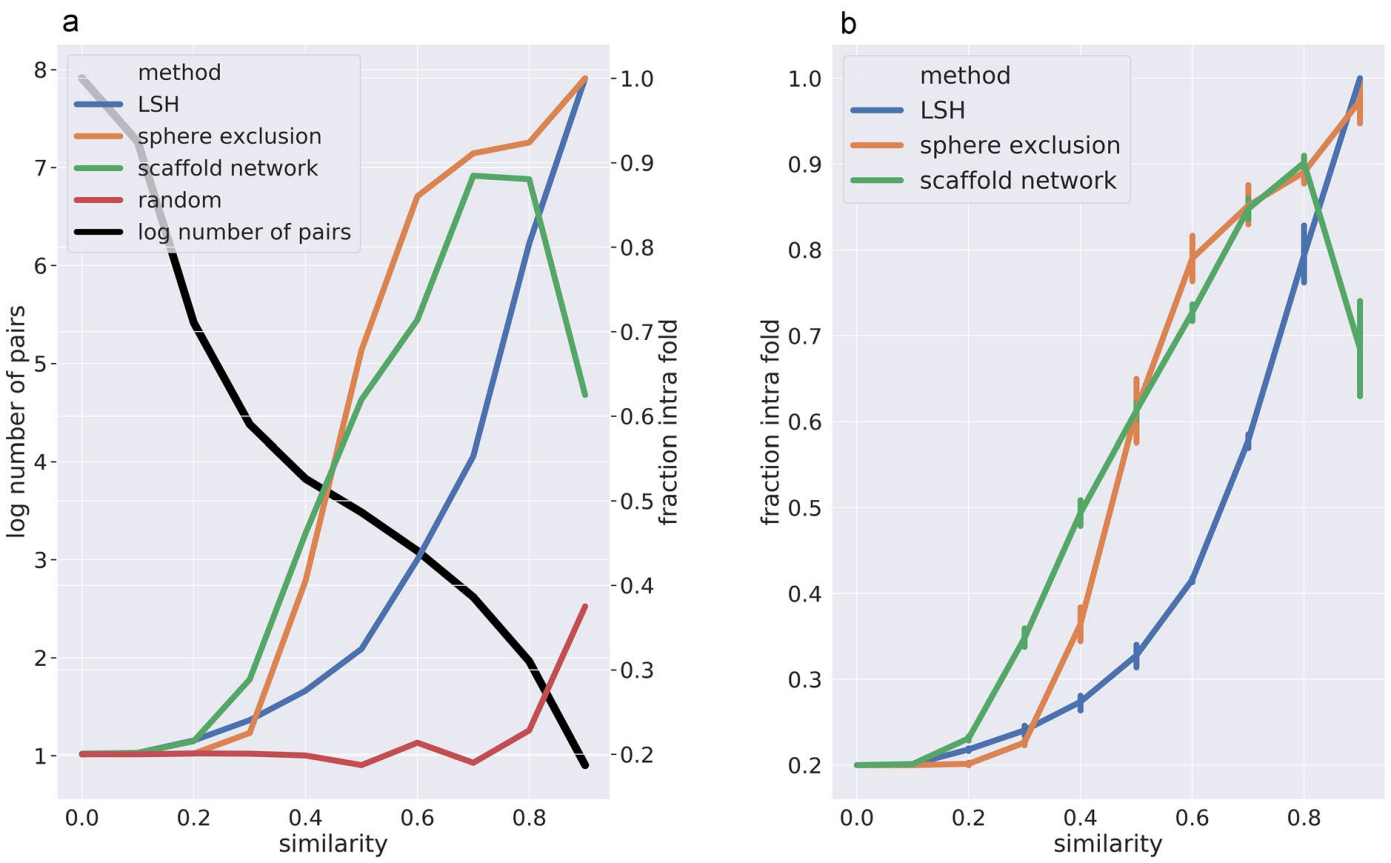
Distribution of compounds over different folds depending on similarity of these compounds. Fraction of intra-fold pairs as function of the Tc ECFP6 similarity of this pair **a** for public data set and **b** averaged over 4 pharma data sets (confidence intervals indicated as bars). In **a** the decadic logarithm of the number of pairs (bold black line) as function of the Tc ECFP6 similarity of this pair is given in addition

A high number ($\sim 10^8$) of random compound pairs were sampled from each partner’s private set and the ChEMBL subset. For each pair, the Tanimoto coefficient was determined and Tanimoto similarities binned. The average fraction of intra-fold (according to LSH, sphere exclusion, and SN) pairs was calculated for each similarity bin. Average fractions over 4 pharma partners are plotted in Fig. 4b. The intra-fold fractions for the public data set as well as the respective number of pairs in each similarity bin are shown in Fig. 4a. In general, for all non-random splits the fraction of intra-fold pairs increases with increasing similarity. For the scaffold-based fold splitting a drop in the intra-fold fraction is observed (Fig. 4) for the highest similarity bin. This can be explained by two facts. First, the sample size for the corresponding bin (0.9-1.0 Tc similarity) is low (see Fig. 4a number of pairs in each bin). Second, small changes in the scaffold can lead to a high similarity based on ECFP6 but different scaffold assignment. The second reason is unique to the scaffold-based folding as LSH as well as sphere exclusion rely on the ECFP6 instead of the scaffold. Four examples of such minor scaffold changes yet maintaining a high ECFP6 similarity are given in Fig. 3. In addition, a slight increase of intra-fold pairs for the highest similarity bin is apparent in random fold splitting (see Fig. 4a). In order to prevent assigning the same compound to different folds, the random folding scheme uses the SMILES for hashing. Hence, identical compounds, which are contained in the highest similarity bin, are assigned to the same fold. Sphere exclusion and scaffold network are detecting subtler similarities than LSH (and random). By assigning even compounds with a low (but apparent) similarity to the same fold, sphere exclusion and scaffold network make it more challenging (i.e. realistic) for the network. This leads to a more realistic estimation of the model’s performance in a real medicinal chemistry application scenario.

### Label and data imbalance

The fraction of tasks below five compounds in one or more folds was used as a measure of task data distribution imbalance. Tasks were binned by size and bin fractions were averaged over four partners and the public ChEMBL subset. Tasks where the fold standard deviation of the fraction of actives was greater than 0.05 were marked as “label imbalanced”. The results of the label and data imbalance analysis are presented in Table 2.

**Table 2:** Label and data imbalance of different folding methods averaged over all tasks of four partners and the ChEMBL subset. Fraction below 05: fraction of tasks below five compounds in one or more folds, fraction label imbalance: fraction of tasks where the fold standard deviation of the fraction of actives was greater than 0.05

Fold method	Task size bin lower limit	Fraction below 05	Fraction label imbalance
LSH	10	0.90	0.35
	100	0.29	0.37
	1000	0.08	0.11
	10000	0.03	0.00
	100000	0.00	0.00
Sphere exclusion	10	0.95	0.46
	100	0.37	0.49
	1000	0.11	0.24
	10000	0.04	0.00
	100000	0.05	0.00
Scaffold network	10	0.96	0.58
	100	0.46	0.64
	1000	0.10	0.29
	10000	0.04	0.08
	100000	0.08	0.12
Random	10	0.67	0.07
	100	0.18	0.05
	1000	0.05	0.00
	10000	0.00	0.00

Although the splitting of the data into test, train and validation set should be as close as possible to a realistic prospective application of the model, enough data including a sufficient amount of all labels in each fold must be given for a sound data basis. All three methods are worse than random with regard to label and data balance for small tasks. LSH performs closest to random but its advantage over sphere exclusion and scaffold network becomes smaller for tasks with 1000 compounds or more. Regarding label and data imbalance all four folding methods are suitable given that the data sets are of reasonable size (more than 1000 compounds).

### Bias in prediction performance

Randomly splitting the data into test, train and validation set clearly gives an overoptimistic view on model performance. Thus, a more realistic splitting will yield in a reduced (less overoptimistic) performance. For each folding method a hyperparameter search using the same grid (see Table 1) and a nested cross-validation was performed. The best hyperparameters were largely consistent for different folding methods (both for pharma and public data sets). For the ChEMBL subset one of two hyperparameter sets (hidden size 1200 or 2000, dropout 0.7 and weight decay 1E-6) was found best for each folding method (see Table 3). The best hyperparameter of each folding method was also always within the top 10 hyperparameters of the other methods. Thus, the hyperparameter preference is not sensitive to the folding method.

**Table 3:** Differences in best hyperparameter selection for different folding methods on the public data set. The top 10 performing hyperparameter sets for the random fold splitting are given together with the respective rank of this setting for the other folding methods

Hidden	Dropout	Weight	Rank	Rank	Rank
Sizes		Decay	Sphere exclusion	Scaffold network	LSH
[2000]	0.7	1E−6	2	1	8
[2000]	0.6	1E−6	8	6	5
[1600]	0.7	1E−6	3	2	2
[1200]	0.5	1E−6	11	13	7
[1200]	0.6	1E−6	9	10	4
[3000]	0.7	1E−6	5	4	6
[1200]	0.7	1E−6	1	3	1
[1600]	0.6	1E−6	6	9	3
[3000]	0.6	1E−6	7	8	11
[1600]	0.5	1E-6	4	11	10

**Fig. 5:**
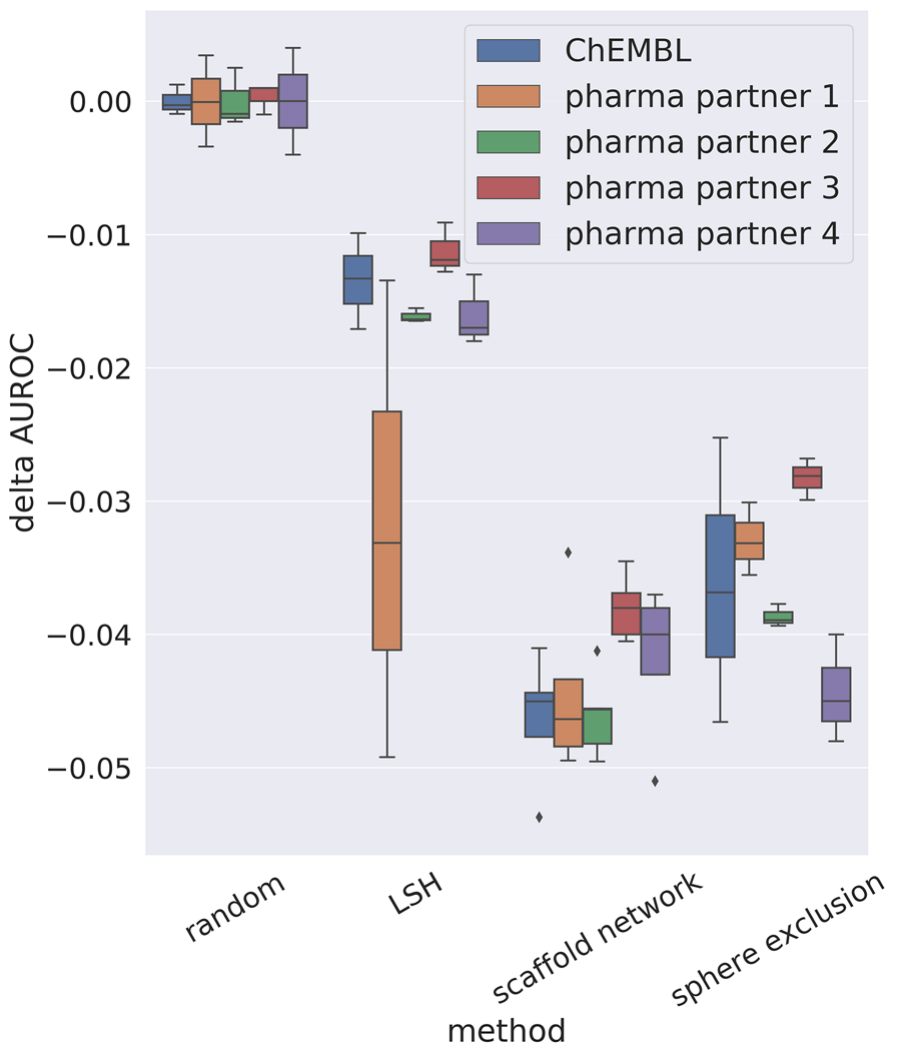
Performance difference of folding methods compared to a random folding. Performance difference by means of delta AUROC averaged over at least three test folds (confidence intervals indicated as bars) and compared to a random folding for four folding methods and all tasks of four partners as well as a ChEMBL subset

We analyzed the performance by means of area under the precision-recall curve (AUPR) and area under the receiver operating characteristic curve (AUROC) and calculated the respective delta performance to the performance of a random splitting. The results are depicted in Fig. 5 and Additional file 1: Fig. S1 for AUROC and AUPR, respectively. Although, AUROC is the more rigorous measure we calculated AUPR in addition, because it is the primary metrics used in MELLODDY.

For all partners and both metrics we observe decreased performance values for all three analyzed folding methods compared to random splitting confirming previous findings that a random fold split leads to overoptimistic performances. Moreover, this indicates that all three methods are able to reduce the overoptimistic performance of a random fold splitting. LSH has the lowest decrease in performance and thus is probably still overoptimistic. A reason could be that the test set contains a higher number of compounds similar to the training set (see Fig. 4b). However, LSH is less biased than random splitting with regards to prediction performance. For all partners but one (pharma partner 1) a clear gap between LSH on the one side and sphere exclusion as well as scaffold network on the other side can be observed. In contrast, the performance gap between sphere exclusion and scaffold network is only marginal. Again for all partners but one (pharma partner 4) scaffold network leads to a slightly larger decrease in performance compared to sphere exclusion. Given that the heuristics underlying the scaffold network have been designed to recover chemical series, it is not surprising that they are effective in suppressing the chemical series effect. Thus, sphere exclusion and scaffold network based splitting are equally well suited to generate proper test, train and validation sets for a more realistic evaluation of model performance.

## Conclusion

Designing proper test, train and validation sets in machine learning is a vital but not trivial task. It is crucial to ensure realistic performance estimates and a fair evaluation of machine learning models for real-world (medicinal chemistry) applications. In this work we studied four train-test splitting methods for chemical structure data: random splitting, locality-sensitive hashing (LSH), scaffold-based binning (scaffold network) and sphere exclusion clustering. To this end, the impact of these four different folding methods was analyzed on four large-scale pharma data sets and a ChEMBL subset and assessed wrt. similarity of the compounds within one fold, label and data imbalance, and predictive performance bias. In addition, we compare these methods regarding their application in a federated privacy-preserving setting which is an attractive opportunity to improve model performance, but leads to restrictions on fold splitting methods. In general the results are comparable between the different data sets indicating a good transferability of the presented results to other data sets. In particular, both sphere exclusion and scaffold network show better enrichment of similar compounds in the same fold and a more realistic performance metrics than LSH. LSH and a random fold splitting distribute compounds and labels more evenly through the folds. This effect vanishes for tasks with 1000 compounds or more. Thus, for a more realistic validation of machine learning models scaffold-based and sphere exclusion fold splitting are beneficial. However, a scaffold-based fold splitting has the advantage that besides allocating similar compounds comparably to sphere exclusion it avoids the necessity of a federated platform level implementation. Hence, scaffold-based fold splitting was implemented and used in the MELLODDY project. Further publications describing the MELLODDY approach in more detail will be published elsewhere. In summary, scaffold-based fold splitting is the preferred fold splitting method for federated privacy-preserving multi-task machine learning models, whereas sphere exclusion clustering is preferred for non-federated settings.

## Supplementary Information

**Additional file 1.** Performance difference of folding methods compared to a random folding. Performance difference by means of delta AUROC and delta area under the precision-recall curve (AUPR) each averaged over at least three test folds (confidence intervals indicated as bars) and compared to a random folding for four folding methods and all tasks of four partners as well as a ChEMBL subset.

## Data Availability

The code and data sets supporting the conclusions of this article are made available at (https://github.com/melloddy/ChemFold) (fold splitting package for machine learning in medicinal chemistry developed as part of this work), 10.5281/zenodo.4778423.(public data set), https://github.com/melloddy/SparseChemh (machine learning package for biochemical applications) and https://github.com/melloddy/MELLODDY-TUNER/tree/release/1.0 (pipeline for data preparation developed as part of the MELLODDY project). As result of the presented work the two fold splitting methods LSH and scaffold-based binning are implemented in the second version of the data preparation pipeline MELLODDY-TUNER https://github.com/melloddy/MELLODDY-TUNER/tree/release/v2.0.

## Footnotes

1 https://github.com/melloddy/MELLODDY-TUNER/tree/release/1.0.

2 https://github.com/melloddy/public_data_extraction.

3 https://github.com/melloddy/MELLODDY-TUNER/tree/release/1.0.

4 10.5281/zenodo.4778423

5 https://github.com/melloddy/SparseChem.
