# Supplementary figures and images for: Splitting chemical structure data sets for federated privacy-preserving machine learning
Source: J Cheminform. 2021 Dec 7;13:96. doi: 10.1186/s13321-021-00576-2 (PMC8650276; doi:10.1186/s13321-021-00576-2)

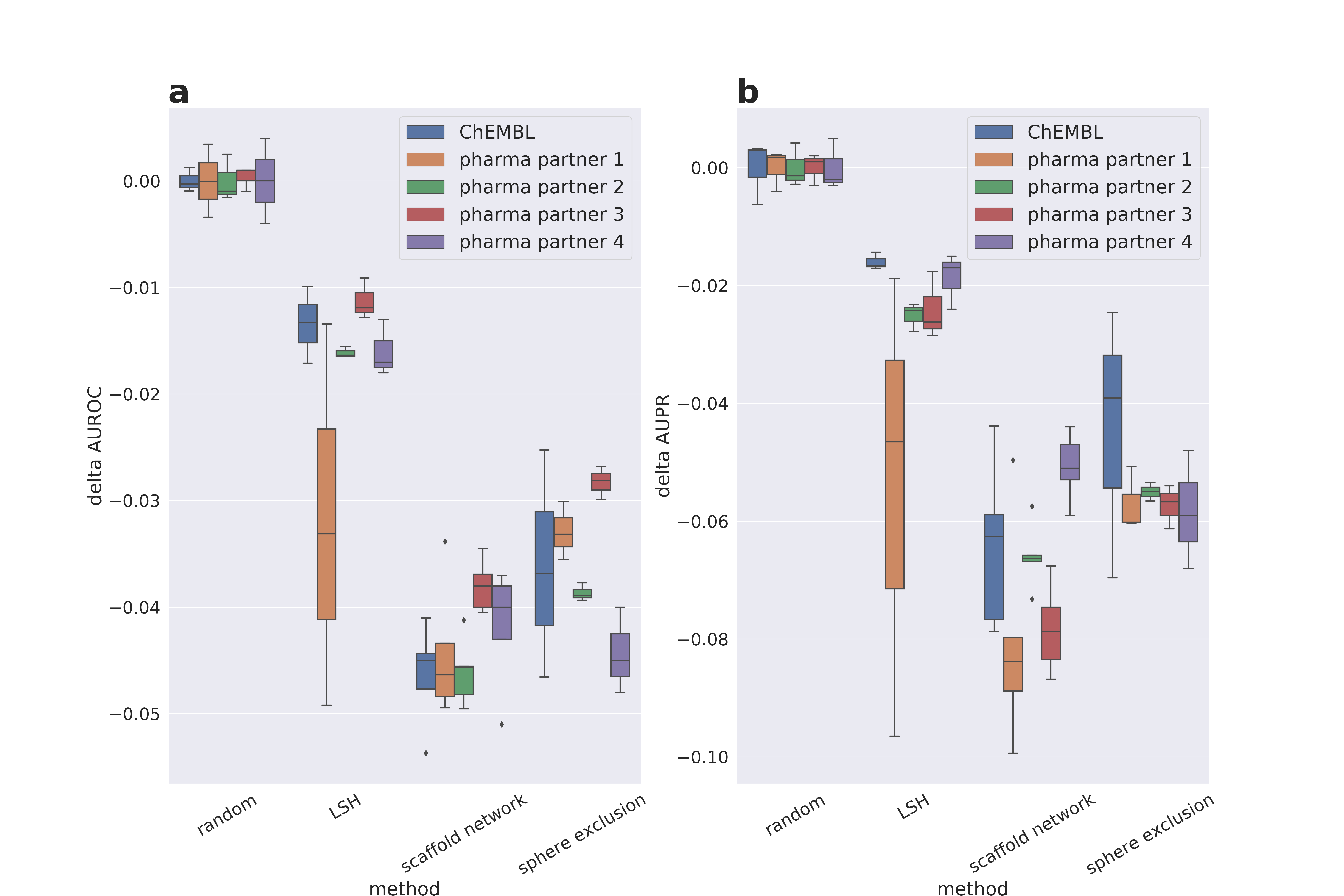

Supplement: Supplementary file 1 — Additional file 1. Performance difference of folding methods compared to a random folding. Performance difference by means of delta AUROC and delta area under the precision-recall curve (AUPR) each averaged over at least three test folds (confidence intervals indicated as bars) and compared to a random folding for four folding methods and all tasks of four partners as well as a ChEMBL subset. [file 13321_2021_576_MOESM1_ESM.png]
